# A Novel Porcine *In Vitro* Model of the Blood-Cerebrospinal Fluid Barrier with Strong Barrier Function

**DOI:** 10.1371/journal.pone.0039835

**Published:** 2012-06-22

**Authors:** Mira Schroten, Franz-Georg Hanisch, Natascha Quednau, Carolin Stump, Roland Riebe, Matthias Lenk, Hartwig Wolburg, Tobias Tenenbaum, Christian Schwerk

**Affiliations:** 1 Pediatric Infectious Diseases, Department of Pediatrics, Medical Faculty Mannheim, Heidelberg University, Mannheim, Germany; 2 Institute of Biochemistry II, Medical Faculty, University of Cologne, Köln, Germany; 3 Friedrich-Loeffler-Institut, Federal Research Institute for Animal Health, Institute of Infectology, Greifswald-Insel Riems, Germany; 4 Institute of Pathology, University of Tübingen, Tübingen, Germany; Biological Research Centre of the Hungarian Academy of Sciences, Hungary

## Abstract

Epithelial cells of the plexus choroideus form the structural basis of the blood-cerebrospinal fluid barrier (BCSFB). *In vitro* models of the BCSFB presenting characteristics of a functional barrier are of significant scientific interest as tools for examination of BCSFB function. Due to a lack of suitable cell lines as *in vitro* models, primary porcine plexus epithelial cells were subjected to a series of selective cultivation steps until a stable continuous subcultivatable epithelial cell line (PCP-R) was established. PCP-R cells grow in a regular polygonal pattern with a doubling time of 28–36 h. At a cell number of 1.5×10^5^ in a 24-well plate confluence is reached in 56–72 h. Cells are cytokeratin positive and chromosomal analysis revealed 56 chromosomes at peak (84th subculture). Employing reverse transcription PCR mRNA expression of several transporters and components of cell junctions could be detected. The latter includes tight junction components like Claudin-1 and -3, ZO-1, and Occludin, and the adherens junction protein E-cadherin. Cellular localization studies of ZO-1, Occludin and Claudin-1 by immunofluorescence and morphological analysis by electron microscopy demonstrated formation of a dense tight junction structure. Importantly, when grown on cell culture inserts PCP-R developed typical characteristics of a functional BCSFB including high transepithelial electrical resistance above 600 Ω×cm^2^ as well as low permeability for macromolecules. In summary, our data suggest the PCP-R cell line as a suitable *in vitro* model of the porcine BCSFB.

## Introduction

The cerebrospinal fluid (CSF) is produced by the choroid plexus (CP), a highly perfused structure located in the ventricles of the brain. In the CP the endothelial cells lining the blood vessels are fenestrated to provide a physiological basis for the CP to produce CSF from the blood. To prevent arbitrary diffusion of blood-borne substances into the brain and to allow regulation of CSF production the epithelial cells of the CP are densely connected by adherens junctions (AJs) and tight junctions (TJs) and thereby constitute the morphological correlate of the blood-CSF barrier (BCSFB). TJs restrict the permeability of e.g. peptides or hydrophilic drugs and also the movement of small ions, thereby leading to formation of a significant transepithelial/transendothelial electrical resistance (TEER) [1]–[3]. Selective import and export from the CP to the CSF and vice versa is mediated by specialized transporter systems [4], [5]. Importantly, BCSFB function can be severely compromised during inflammatory events in the central nervous system (CNS) [6].

Due to these fundamental functions *in vitro* models of the BCSFB are of high interest for basic as well as industrial research studying the role of the CP epithelium in CNS pharmacology, neurological disorders and during infectious diseases of the brain [7]. A primary culture model of the BCSFB with excellent barrier functions consisting of primary porcine choroid plexus epithelial cells (PCPEC) has been established [8], [9], which allows analysis of the interaction with bacterial pathogens in the physiologically relevant basolateral-to-apical direction [10]. However, contrary to primary culture systems, the establishment of CP cell lines offers many advantages for experimental studies such as low costs, easy cultivation and the option of genetic manipulation. Several CP cell lines originating from murine [11]–[13] or human [14], [15] systems have been described (a comprehensive recent review covering BCSFB *in vitro* models is given by Strazielle and Ghersi-Egea [7]. These cell lines differ in expression of tight junction proteins, transport proteins and the development of TEER. Noteworthy, of those analyzed so far, the above-mentioned cell lines all do not present continuous TJ strands and develop only a rather low TEER, thereby limiting their suitability as adequate models of the BCSFB [16], [17].

Based on PCPEC cultures [8], [9] we have established a porcine choroid plexus epithelial cell line (PCP-R) and analyzed the expression of tight junction proteins, transport proteins and the development of TEER as well as the permeability of macromolecules. We demonstrate that PCP-R form tight junctions and display important characteristics of a functional BCSFB *in vitro* model, i.e. the development of a high TEER concomitant with a low permeability for macromolecules when grown on cell culture inserts.

## Results

### Establishment of a stable porcine choroid plexus epithelial cell line (PCP-R)

**Figure 1:**
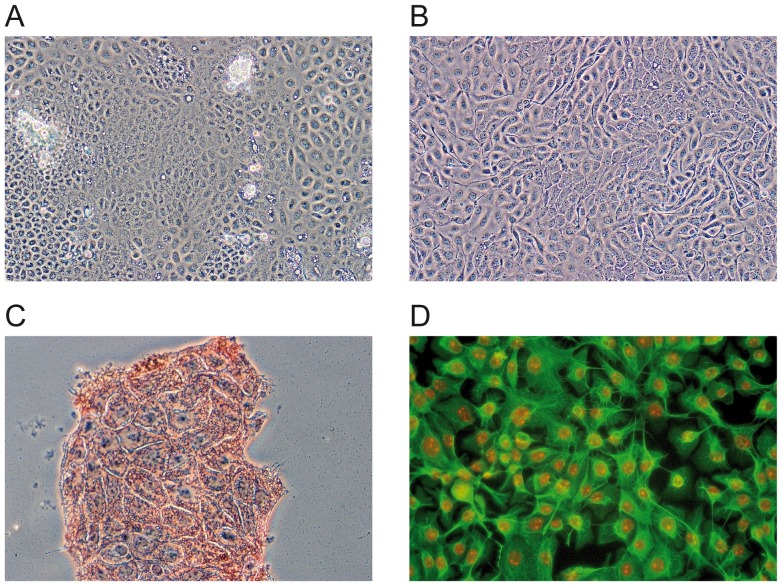
Generation of a stable subcultivatable porcine choroid plexus cell line. (A) Light microscopic depiction of primary porcine choroid plexus epithelial cells. (B) Subculture 43 (day 4): pure population of epithelial choroid plexus cells. These cells were continuously subcultivatable. (C) Immunocytochemistry with a mouse-anti human cytokeratin monoclonal antibody. PCP-R express the epithelial cell marker cytokeratin. (D) Immunofluorescence analysis of PCP-R shows expression of cytokeratin (green). The nuclei are counterstained with propidium iodide (red). Subculture 40 was analyzed in (C) and (D).

Previously, PCPEC have been established as a primary porcine model of the BCSFB [8], [9]. To be able to gain benefit of the advantages a subcultivatable cell line would offer we set out to generate a stable cell line from PCPEC. When subject to serial subcultivation the primary PCPEC culture (Fig. 1A) was displaced by fibroblastoid cell types after the fourth passage. To establish a stable CP epithelial cell line the primary culture was subjected to a series of sequential selection steps, which are described in detail in the “Experimental procedures”.

Fig. 1B shows subculture 43 (after 4 days) as described in “Experimental procedures” of a pure population of epithelial CP, which were continuously subcultivatable. These cells were all positive for mouse-anti human cytokeratin monoclonal antibodies (Fig. 1C, D) as well as negative for von Willebrand factor, pointing to absence of endothelial cells [18] (data not shown). We termed this stable subcultivatable cell line PCP-R.

**Figure 2:**
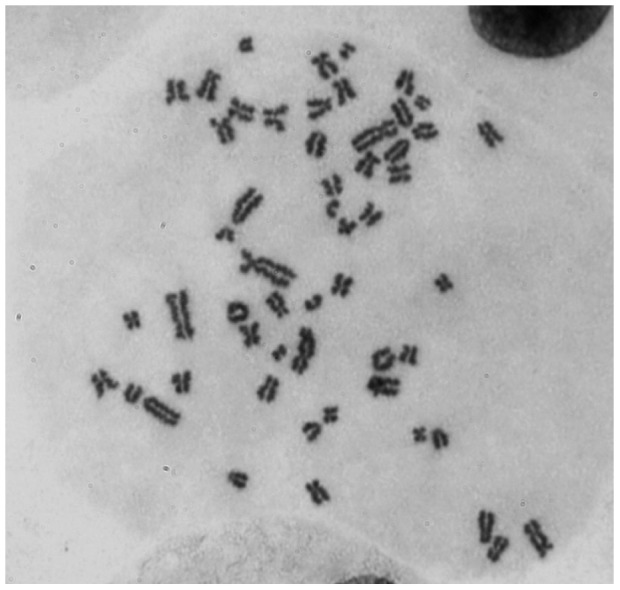
Chromosomes of PCP-R. Chromosome analysis revealed aneuploidy with 56 chromosomes at peak.

We further determined the stem-line of the PCP-R cells. The normal diploid chromosome set in porcine cells contains 38 chromosomes. Chromosome analysis of PCP-R (Fig. 2) revealed in the 84^th^ subculture 56 chromosomes at peak and showed aneuploidy, a phenomenon, which is known for spontaneous immortalized porcine cells [19].

### Transporter expression in PCP-R

An important feature of the BCSFB is the presence of transporter proteins, which mediate selective substance exchange between blood and CSF [4], [5]. To gain information regarding transporter expression in PCP-R we performed reverse transcriptase polymerase chain reactions (RT-PCRs) on RNA sampled from PCP-R. Employing qualitative RT-PCR we detected expression of mRNAs encoding for the transporter proteins divalent metal transporter-1 (DMT-1), transferrin receptor (TFRC), zinc transporter-1 (ZnT1), leptin receptor (LEPR) and ATPase 7A (ATP7A) in the PCP-R cells (data not shown), which have previously been described to be expressed in the rat CP cell line Z310 [20]. We further compared the relative mRNA expression levels of the transporters with those observed in whole porcine CP tissue by quantitative real-time PCR (QPCR) after normalization of the data to β-actin in the same samples. Whereas expression of TFRC was similar, relative mRNA levels of DMT1, ZnT1 and ATP7A were statistically significantly lower in the PCP-R (Table 1). Expression of LEPR was extremely low in PCP-R compared to whole CP tissue (data not shown).

**Table 1:** Comparison of mRNA expression levels of genes encoding for transporter proteins and junctional proteins in PCP-R and porcine CP tissue.

gene	Porcine CP tissue	PCP-R
Claudin-1	100±15.7	39.3±7.4**
Claudin-3	100±12.5	5.8±3.7***
ZO-1	100±31.6	20.5±12.3*
Occludin	100±13.9	34.9±10.1**
E-cadherin	100±4.8	3351.8±1464.5
DMT-1	100±18.4	2.4±0.8*
TFRC	100±27.2	73.8±74.9
ZnT1	100±27.1	5.7±4.1*
ATP7A	100±22.7	4.3±1.8*

The mRNA expression of all analyzed genes was normalized that of β-actin in the same sample. The expression in porcine CP tissue was arbitrarily set as 100%. Data represent mean ± SD (n = 3).

* p<0.05.

** p<0.01.

*** p<0.001 as compared to controls.

### PCP-R express junction proteins and develop morphologically dense tight junctions

Typically, CP epithelial cells express AJ and TJ proteins to mediate cellular polarization and regulation of barrier function [3], [21]. To investigate the presence of AJ and TJ components in PCP-R we determined expression of a typical AJ protein (E-cadherin) and of several TJ associated factors (Claudin-1, -2, -3, ZO-1, Occludin) by RT-PCR on RNA samples from PCP-R. Qualitative RT-PCR analysis of PCP-R revealed that all investigated AJ and TJ components are expressed on RNA level in PCP-R cells (data not shown). As with the transporter mRNA analysis we compared the relative mRNA expression levels of the junctional proteins with those observed in whole porcine CP tissue by quantitative real-time PCR (QPCR) after normalization of the data to β-actin in the same samples. As can be seen in Table 1, whereas expression of E-cadherin was similar, relative mRNA levels of Claudin-1, Claudin-3, ZO-1 and Occludin were statistically significantly lower in the PCP-R. Expression of Claudin-2 was extremely low in PCP-R compared to whole CP tissue (data not shown).

**Figure 3:**
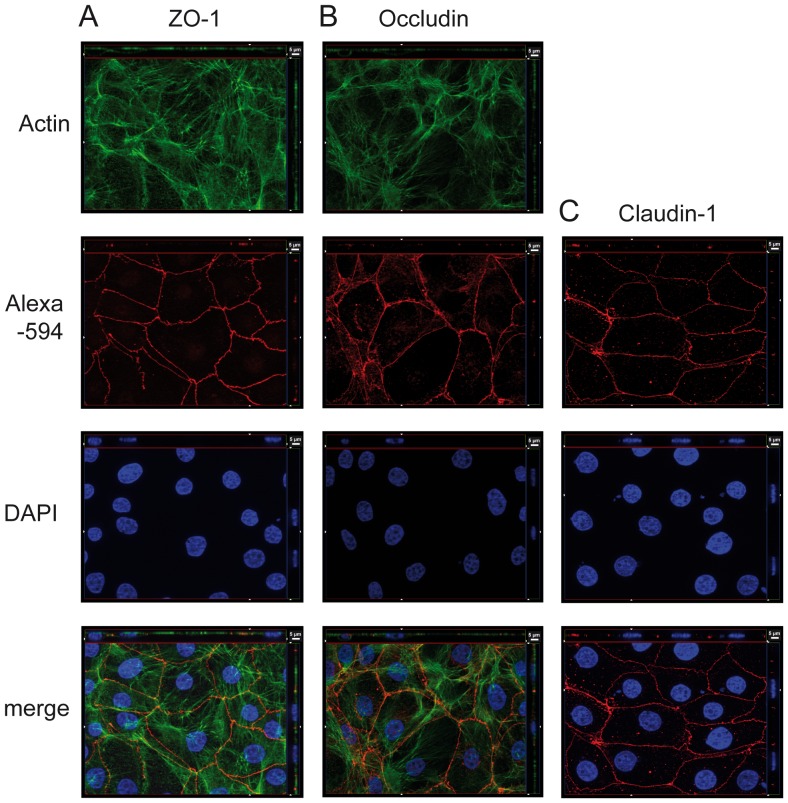
PCP-R displayed continuous tight junction strands. The cells, grown on coverslips, were stained for detection of ZO1 (A), Occludin (B) and Claudin-1 (C). Secondary antibodies were labeled with Alexa Fluor 594 (Alexa-594, red). Pictures presented are Apotome-generated images; *bottom* of each panel is an *xy* en face view of a cell culture monolayer shown in a maximum-intensity projection through the z-axis; *top and side* of each panel is a cross section through the z-plane of multiple optical slices. The basolateral side of PCP-R is oriented towards the top or right side, respectively, of the top and right images of each panel. In (A) and (B) the actin cytoskeleton was in parallel stained with phalloidin Alexa Fluor 488 (green). Since Claudin-1 samples were fixed with methanol we could not observe a qualitatively sufficient actin staining. In all samples nuclei were stained with DAPI (blue). The images shown are representative examples of multiple stainings.

To gain insight regarding the tight junction morphology of PCP-R we analyzed cell layers grown on coverslips by immunofluorescence-staining against ZO-1, Occludin and Claudin-1 (Fig. 3). In agreement with the RT-PCR data, all three investigated proteins were expressed and could be detected on protein level by immunofluorescence microscopy. The *xy* en face view of the Apotome images revealed the monolayer structure of the confluent PCP-R cells. All three investigated TJ-associated factors displayed a continuous pattern localized apically at the sites of cell-cell contact (Fig. 3).

**Figure 4:**
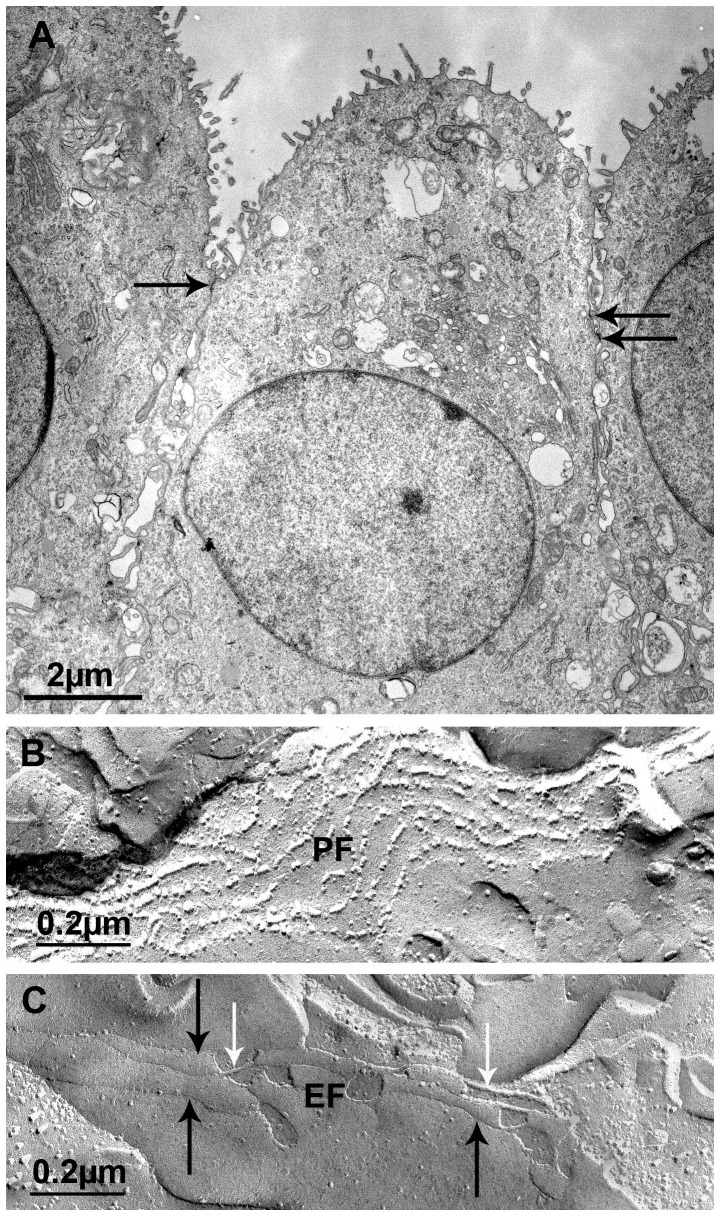
Electron microscopic analysis of PCP-R. (A) Ultrathin section through a monolayer of porcine choroid plexus epithelial cells cultivated on polycarbonate filters with pores. The cells contain a round nucleus with much euchromatin, at the apical pole the cells develop numerous, but short, microvilli, and the cells are interconnected by tight junctions (labeled by arrows), which are located directly below the apical microvilli. This culture developed an electrical resistance of 563 Ω×cm^2^. (B) Freeze-fracture replica of porcine choroid plexus cells showing tight junctions, which mainly were characterized by parallel and highly P-face (PF) associated strands. This culture developed an electrical resistance of 649 Ω×cm^2^. (C) Freeze-fracture replica of porcine choroid plexus epithelial cells showing tight junction strands at the P-face (white arrows) and at the E-face (black arrows). At the E-face, tight junctions can be observed as widely particle-free grooves. The replicas shown in (B) and (C) were taken from the identical culture.

For a most detailed morphological impression we analyzed PCP-R filter insert cultures with a TEER around 600 Ω×cm^2^ by transmission as well as freeze fracture electron microscopy (Fig. 4). Transmission electron microscopic images reveal the presence of TJs between the cells close to the apical side, which contains numerous short microvilli. Furthermore, the cells contain a round nucleus with much euchromatin (Fig. 4A). Freeze fracture studies of PCP-R show mainly parallel, P-face associated TJs (Fig. 4B). At the E-face, TJs are widely particle-free grooves (Fig. 4C).

### PCP-R develop a barrier function on cell culture inserts

**Figure 5:**
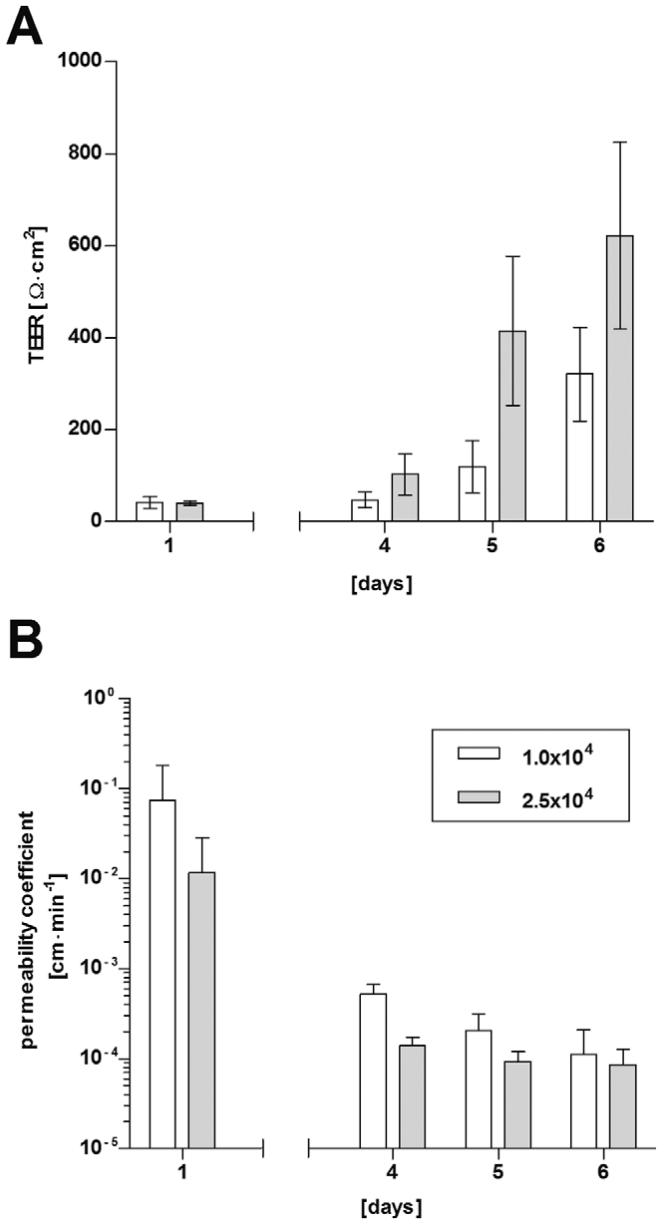
PCP-R display barrier functions when grown on cell culture inserts (high TEER values correlate with low FITC-inulin flux through PCP-R-layers). 1.5×10^4^ (white bars) or 2.5×10^4^ (grey bars) PCP-R cells were seeded on cell culture inserts; TEER values (A) and permeability coefficients for FITC-inulin (B) were measured over time. Shown is the mean ± SD of at least three experiments performed in duplicates.

An efficient model of the BCSFB is characterized by development of a high TEER and low paracellular permeability of macromolecules [7]. To determine the applicability of PCP-R as model system for the BCSFB according to these parameters we grew PCP-R on cell culture inserts and measured the TEER values as well as the flux of FITC-labeled inulin (FITC-inulin) over time. As can be seen in Fig. 5A PCP-R developed a high TEER, which, dependent on the number of cells seeded onto the filter membrane (1×10^4^ or 2.5×10^4^ cells) increases from day one of cultivation (42 Ω×cm^2^ or 39 Ω×cm^2^) to 321 Ω×cm^2^ or 621 Ω×cm^2^, respectively, on day 6 (Fig. 5A).

Another typical hallmark of the functional BCSFB is a low permeability for macromolecules reciprocal to a high TEER [8], [9]. We therefore measured the FITC-inulin flux through PCP-R layers grown on cell culture inserts in parallel to TEER development and calculated the permeability coefficients in cm min^−1^. As demonstrated in Fig. 5B permeability coefficients of PCP-R layers dropped from above 1.0×10^−2^ cm min^−1^ (1.2×10^−2^ to 7.5×10^−2^ cm min^−1^, dependant on cell amount seeded initially) on day 1 when TEER values are still low to around 1.0×10^−4^ cm min^−1^ at day 6, simultaneously with the development of a high TEER.

## Discussion

The epithelial cells of the CP are responsible for barrier function of the BCSFB. To study the roles of the BCSFB during diverse cellular processes *in vitro* models recapitulating the characteristic properties of the BCSFB are of fundamental scientific interest. Here we employed primary porcine choroid plexus epithelial cells (PCPEC) [8], [9] to establish a novel stable porcine choroid plexus epithelial cell line (PCP-R) by spontaneous immortalization. PCP-R displayed an epithelial morphology, were cytokeratin positive and negative for von Willebrand factor, pointing to absence of endothelial cells [18]. A chromosomal analysis revealed a stemline of 56 chromosomes at peak in the 84^th^ subculture.

Functionally relevant *in vitro* models of the BCSFB need to offer proper barrier functions, which largely depend on the formation of intercellular junctions [7]. Of the described CP epithelial cell lines several have been analyzed regarding the presence of AJs and TJs. These include the rat CP epithelial cell lines Z310 and TR-CSFB3, which have been established using transfection with the simian virus 40 large T-antigen [11]–[13], as well as the human cell line CPC-2 derived from CP carcinoma [14]. Szmydynger-Chodobska et al. [16] investigated the expression of junctional proteins in these three cell lines. In all cell lines the expression of the AJ component E-cadherin was found on mRNA and protein level, but E-cadherin staining was discontinuous in TR-CSFB3 and mislocalized in CPC-2 cells. Analysis of TJ proteins revealed expression of Occludin on mRNA and protein level in all three cell lines, but again a continuous staining pattern was only found for Z310. Staining of the TJ-associated protein ZO-1, which was found in both rat cell lines, but not in CPC-2 cells, was mislocalized in TR-CSFB, too. Transcripts of different Claudins were found in TR-CSFB3 and CPC-2 cells, but only Claudin-1 was found on protein level and was either discontinuous (TR-CSFB3) or mislocalized in the nucleus (CPC-2) [16]. In Z310 cells expression of Claudins seems to be absent or very low compared to primary cells [12], [16].

In our study PCP-R cells expressed E-cadherin, Occludin, ZO-1 and Claudins on mRNA level, which to a large extend recapitulates the expression of junctional proteins as described for the primary PCPEC [8], [9], [22]. Noteworthy, the relative mRNA expression levels of Claudin-2 in PCP-R was very low compared to whole porcine choroidal tissue. This stands in contrast to the described expression of Claudin-2 in mouse CP epithelium [23], indicating a loss of certain characteristics and possibly properties of CP epithelial cells in PCP-R. Immunofluorescence analyses revealed that Occludin, ZO1 and Claudin-1 are all located correctly and in a continuous manner at the cell-cell borders. So far we did not investigate further junctional proteins on protein level. The presence of continuous tight junctions between PCP-R cells was further confirmed by transmission and freeze-fracture electron microscopical studies. Concerning other described CP cell lines, a “TJ” type of structure was found by transmission electron microscopy in the sub-apical lateral membranes between adjacent Z310 cells [20]. Additionally, Kläs et al. [17] have looked for tight junctions in Z310 and TR-CSFB cell lines using freeze-fracture electron microscopy. Although expression of TJ proteins could be improved by steroid treatments, continuous TJ strands or networks were never found [17].

Concomitant with morphologically correct TJs the development of a high TEER has been stated as characteristic for efficient models of the BCSFB [7]. PCP-R display TEER values above 600 Ω×cm^2^, which is in the order of magnitude of those obtained with PCPEC [8], [9]. For both PCP-R and PCPEC the high electrical resistance agrees with the presence of intact TJ strands. Noteworthy, these TEER values are considerably higher than those described for other CP cell lines. Kitazawa et al. [11] reported a net membrane resistance of 50 Ω×cm^2^ for TR-CSFB3 cells. For the Z310 cell line initially TEER values varying between 150–200 Ω×cm^2^ [13] and around 60 Ω×cm^2^, which could be increased by 50% after dexamethasone treatment [12], have been described. In a recent publication by Kläs et al. [17] both Z310 and TR-CSFB reached a TEER of 30–40 Ω×cm^2^, which could not be elevated after steroid treatment. The low TEER values obtained with Z310 und TR-CSFB cells are in agreement with their lack of continuous TJ strands. Since it has been shown that expression of Claudin-2 in epithelial cell lines leads to decreased TEER values [24], the high TEER in PCP-R could also be related to their reduced expression of Claudin-2.

Simultaneously to the development of a high TEER the paracellular permeability of PCP-R for FITC-inulin drops to minimal levels as measured by the flux of FITC-inulin, an observation that was also made with PCPEC [10]. Permeability coefficients for PCP-R filter insert cultures drop to levels of about 1.0×10^−4^ cm min^−1^. If this effect is necessarily due to a direct link between the measured TEER and paracellular permeability in PCP-R is not clear. E.g. the epithelial cell line MDCK I presents a reduced TEER when overexpressing Claudin-2, but the paracellular permeability is unchanged [24]. Along these lines, the permeability coefficient of PCP-R, although it does not reach the quality of primary cultures of porcine or murine CP epithelial cells [7], is comparable to that of inulin determined for Z310 cells by Shi et al. [20], which display lower TEER values than PCP-R. In any case, the minimal levels of paracellular permeability exhibited by PCP-R support their barrier qualities. Noteworthy, Kitazawa et al. have noted that TR-CSFB3 cells, when cultured on cell culture inserts, do not allow reliable transcellular studies [11].

Since the CPs are responsible for substance exchange between the blood and the CSF the CP epithelial cells need to express specific transporter systems [4], [5]. A number of transporters have been reported to be present in Z310 and TR-CSFB3 cells [17], [20], [25]. In a preliminary analysis by RT-PCR and QPCR we could detect the expression of DMT-1, TFRC, ZnT1, ATP7A and LEPR, although the expression of LEPR was extremely low compared to porcine CP tissue. Except for TFRC, the metal transporters and metal carrier protein transporters analysed in this study are expressed significantly lower when compared to CP tissue, which is not the case most of the equivalent transporters in Z310 cells [20]. Since different expression levels of these transporters as well as LEPR might limit the use of PCP-R for transport studies, further experiments will be necessary not only to determine the presence of additional transporters in PCP-R, but also to compare the transport kinetics of molecules (e.g. peptides, nucleotides, nucleosides, metal ions) corresponding to the single transporters between the PCP-R cell line and PCPEC.

In conclusion, we have established PCP-R as novel porcine CP epithelial cell line, which displays important features of a functional BCSFB *in vitro* model, i.e. the formation of a strong barrier function as demonstrated by a high TEER and a low permeability for macromolecules.

## Experimental Procedures

### Establishment and cultivation of a porcine choroid plexus epithelial cell line (PCP-R)

Porcine plexus epithelial cells were isolated as described previously [8], [9]. In order to suppress the high proliferation rate of fibroblasts contained in the primary culture the cells were treated with Ara-C (cytosine arabinoside) according to a scheme published by Wood [26], combined with a fractionated trypsinization. Ara-C is known as a potent suppressor of fibroblast growth and was used to generate fibroblast-free cell cultures. Cells were treated for a week in intervals with 10^−5^ M Ara-C (listed in Table 2). Because of faster detachment of fibroblasts in trypsin-EDTA solution, we also applied a fractionated trypsinization. The fractionated trypsinization was performed when fibroblasts were present in the cell culture. Fibroblasts were removed from the culture after short exposure to 0.5 g l^−1^ trypsin/0.2 g l^−1^ EDTA (Biochrom, Berlin, Germany). Small islets of epithelial-like cells remained in the culture vessels. Their proliferation started about 4 weeks later. No cloning of cells occurred during these steps. While treated with Ara-C the more proliferative fibroblasts are inhibited. Dense cultures were splitted for subculturing. Culturing was done in a mix of Iscove's MDM and Ham's F12 containing 5 µg ml^−1^ insulin, 10% fetal calf serum (FCS) while selecting epithelial-like cells and in the following 40 subcultures. After these subcultures the cells were well characterized by us, cultures with a high ratio of epithelial cells are found from subculture 8 on.

**Table 2:** Scheme for treatment of the cell cultures with Ara-C

Days	Treatment
1, 3, 5, 8	10^−5^ M Ara-C
2, 4, 6, 7, 9, and following days	no Ara-C

Ara-C, cytosine arabinoside.

**Table 3:** Synthetic oligonucleotides used for RT- PCR and QPCR

Gene symbol	Primer	Sequence (5′>3′)	Ref.
CLDN1	forward	CCTACGCTGGTGACAACATTG	This study
CLDN1	reverse	CATTCATGCCAACAGTGGC	This study
CLDN2	forward	CTCGTTGGCCTGTATCATCACC	[27]
CLDN2	reverse	CAGGGGGGAGTAGAAGTCCC	[27]
CLDN3	forward	AACACCATCATCCGGGACTTC	This study
CLDN3	reverse	CGCGGAGTAGAGGATCTTGG	This study
ZO-1	forward	CCTGCTTCTCCAAAAACTCTT	This study
ZO-1	reverse	TTCTATGGAGCTCAACACCC	This study
OCLN	forward	ACGAGCTGGAGGAAGACTGGATC	This study
OCLN	reverse	CCCTTAACTTGCTTCAGTCTATTG	This study
CDH1	forward	CCCTGCCAATCCTGATGA	This study
CDH1	reverse	GCCCCACTCGTTCAGGTA	This study
DMT1	forward	TCGCCAACGGGATAGGC	This study
DMT1	reverse	GATGCTTACCGTGTGCCCA	This study
TFRC	forward	CCCTCGTGAAGCTGGATCT	This study
TFRC	reverse	CTGTATGCCACGTAACCCTCA	This study
ZnT1	forward	TACATGCAGGTGGCTAAGACC	This study
ZnT1	reverse	TGTCCCACAACATTGCTTCAAA	This study
LEPR	forward	TGCCACCAAATACAACATATGACTTC	This study
LEPR	reverse	TCCTCACTCCAAAAGCAACAGTG	This study
ATP7A	forward	ACGGATATCCCTTGCAAATGGC	This study
ATP7A	reverse	CCTCCTTGTCATGAACTGGTGT	This study
ACTNB	forward	TCCAGAGGCGCTCTTCCA	[28]
ACTNB	reverse	CGCACTTCATGATCGAGTTGA	[28]

ACTNB, β-actin; ATP7A, ATPase 7A; CDH1; E-cadherin; CLDN, claudin; DMT1, divalent metal transporter-1; LEPR, leptin receptor; OCLN, Occludin; TFRC, transferrin receptor; ZnT1, zinc transporter-1; ZO-1, tight junction protein ZO-1.

For experiments, PCP-R cells were cultured in DMEM containing 4.5 g l^−1^ glucose, 12 ml penicillin (100 U ml^−1^) and streptomycin (100 µg ml^−1^), 10% heat inactivated FCS, 24 mM HEPES (pH 7.0) and 5 µg ml^−1^ insulin (PCP-R medium). For cell culter insert-based studies cells were seeded in the upper filter well of filter supports (pore diameter 0.4 µm, pore density 1.0×10^8^ pores per cm^2^, 0.33 cm^2^; Greiner Bio-One, Frickenhausen, Germany). On the first day cells were washed with PCP-R medium and PCP-R medium was added to the lower well. On day 4 and day 6 PCP-R medium was changed by moving filters to a new PCP-R medium-flooded 24-well plate and adding new PCP-R medium in the upper filter.

### Chromosome analysis

The cells were treated with 0.1% trypsin solution for 15 seconds at room temperature, stained with 3% Giemsa (pH 6.8), and analyzed for G-band karyotyping. Histograms of chromosome number distribution were determined from 50 metaphases.

### Measurement of TEER

The TEER of PCP-R grown on cell culture inserts was measured with an epithelial tissue voltohmmeter using the STX-2 electrode system (Millipore, Schwalbach, Germany).

### Determination of paracellular permeability and calculation of permeability coefficients

For the investigation of paracellular permeability the passage of a FITC-inulin (Sigma, Deisenhofen, Germany) tracer solution (100 µg ml^−1^, average molecular weight, 3000–6000) from the apical to the basolateral compartment of cell culture inserts over a period of 2 h was measured in a Tecan Infinite M200 Multiwell reader (Tecan, Switzerland) at an extinction of 485 nm and an emission of 535 nm using iControl software. Permeability coefficients of the PCP-R cultures were calculated as described by Shi et al. [20].

### Light microscopy

All light microscopy was done with a Nikon diaphot 300. Photography was done with a Canon EOS D60.

### Electron microscopy

For transmission electron microscope analysis PCP-R cells were grown on cell culture inserts with a pore size of 0.4 µm. Filters with a TEER around 600 Ω×cm^2^ were washed with PCP-R medium and then fixed for 4 h using a 2% glutaraldehyd solution in 75 mM cacodylatbuffer (pH 7.4). Subsequently, the cells were washed twice with 75 mM cacodylatbuffer (pH 7.4) and the support films were removed with a sharp ophthalmic scalpel. The filters were then cut into stripes and postfixed in 1% osmium tetroxide (OsO_4_) in cacodylate buffer for 1 h and dehydrated in ascending series of ethanol and propyleneoxide. For contrast enhancement, they were bloc-stained in uranyl-acetate in 70% ethanol for 4 h and flat-embedded in Araldite (Serva, Heidelberg, Germany). Using an ultramicrotome (Ultracut R, Leica, Bensheim, Germany), semi- (1 µm) and ultrathin sections (50 nm) were cut. Ultrathin sections were stained with lead citrate, mounted on copper grids and finally analysed with a Zeiss EM 10 (Oberkochen, Germany) electron microscope.

For freeze-fracturing, the filter stripes were treated with 30% glycerol and quick-frozen in nitrogen-slush (−210°C). Subsequently, the specimens were fractured in a Balzer's freeze-fracture device (BAF400D; Balzers, Liechtenstein) at 5×10^−6^ mbar and −150°C. Both complementary fracture faces were shadowed with platinum/carbon (2 nm, 45°) for contrast and carbon (20 nm, 90°) for stabilization of the replica. After removing the cell material in 12% sodium hypochlorite, the replicas were cleaned several times in double-distilled water and mounted on Pioloform-coated copper grids. The replicas were observed using an EM10A electron microscope (Carl Zeiss, Oberkochen, Germany). The pictures of ultrathin sections and freeze-fracture replicas were scanned at 300 dpi and processed with Adobe Photoshop.

### Immunohistochemistry and immunofluorescence

To verify absence of endothelial cells in the given cell population of PCP-R the cells have been checked with indirect immunofluorescence for absence of von Willebrand Factor as a marker of endothelial cells [18]. The cells were grown on coverslips, washed in PBS, fixed in acetone for 10 min at room temperature, washed again in PBS, and incubated 1 hour at room temperature with 1∶200 diluted primary antibody (polyclonal rabbit anti-human von Willebrand factor (DakoCytomation, Glostrup, Danmark); the antibody shows cross-reactivity in swine endothelial cells). Subsequently, the coverslips were washed 5 times with PBS for 5 min and incubated 1 hour at room temperature with a 1∶200 dilution of the secondary antibody (Alexa Fluor 488 goat anti-rabbit IgG (H+L) (Molecular Probes, Leiden, Netherlands)). Afterwards, coverslips were again washed 5 times with PBS for 5 min and mounted in polyvinyl alcohol mounting medium with DABCO (Fluka, Sigma-Aldrich, Deisenhofen, Germany) containing propidium iodide (adjusted to 1 µg ml^−1^; Molecular Probes, Oregon, USA) to counterstain nuclei. Cytokeratin staining was done in the same way. The primary monoclonal antibody was mouse anti-human cytokeratin, PAN (1∶200 dilution; Serotec, Kidlington, UK). As secondary antibody Alexa Fluor 488 goat anti-mouse IgG (H+L) (1∶200 dilution; Molecular Probes, Oregon, USA) was used. Microscopy was done using a Nikon diaphot 300, pictures were taken with a Canon EOS D60.

For visualizing the cytoskeleton as well as tight junctions, PCP-R were grown on coverslips until confluence did occur on day three. Then cells were washed with phenolred-free medium and fixed with 4% formaldehyd for 15 min at room temperature. Ice-cold 100% methanol was used for claudin-1 visualization and was applied for 20 min at 4°C. Afterwards coverslips were washed five times with PBS and permeabilized for 60 minutes at room temperature applying PBS containing 0.5% Triton and 1% BSA (bovine serum albumin). Coverslips were then incubated with primary antibodies over night in a 1∶250 dilution (rabbit anti-Occludin 0.25 µg ml^−1^; rabbit anti-Claudin-1 0.25 µg ml^−1^; rabbit anti-ZO-1 0.25 µg ml^−1^ (all antibodies from Zymed, San Francisco, USA)). The next day coverslips were washed three times with PBS and subsequently the following secondary fluorophor-labelled antibodies in PBS/1% BSA were added for 60 min at room temperature: chicken anti-rabbit Alexa Fluor 594 (1∶250 dilution; Molecular Probes, Oregon, USA). The cytoskeleton was stained with phalloidin Alexa Fluor 488 (1 U 300 µl^−1^; Molecular Probes, Oregon, USA), nuclei were stained with 4′-6-diamidino-2-phenylindole dihydrochloride (DAPI) (1∶50.000). Images were acquired with Zeiss Apotome and Axiovision Software (Carl Zeiss, Jena, Germany) using a 63×/1.4 objective lens. This system provides an optical slice view reconstructed from fluorescent samples. Fig. 4 shows a representative selection of images chosen from multiple standard microscopic fields. All immunofluorescence experiments were repeated at least three times.

### Reverse-transcriptase polymerase chain reaction

For quantitative real-time reverse-transcriptase polymerase chain reactions (QPCR) PCP-R cells (passages 55 to 60) were grown in PCP-R medium until confluency. Subsequently, cells were cultured for on additional day in PCP-R medium containing 10% FCS. The RNA of PCP-R, was isolated using the RNeasy mini kit (Qiagen, Hilden, Germany). Afterwards 1 µg of total RNA was transcribed into cDNA with the Affinity Script QPCR cDNA synthesis kit (Agilent Technologies, Santa Clara, CA) according to the manufacturer's instructions. For preparation of mRNA from isolated porcine choroidal tissue, brains from freshly slaughtered pigs were dissected and the CP tissue from the lateral and the fourth ventricles was removed and washed with twice with HBSS (no Ca^2+^, no Mg^2+^) containing 24 mM HEPES (pH 7.0) and 10 ml penicillin (100 U ml^−1^) and streptomycin (100 µg ml^−1^). Isolation of RNA from porcine Plexus was performed with the TRIzol Reagent (Invitrogen, Karlsruhe, Germany). RNA was converted into cDNA with an oligo-dT primer using the AffinityScript QPCR cDNA Synthesis Kit (Stratagene, La Jolla, CA) following the instructions provided by the manufacturer. Subsequently, QPCR was performed in an MX3005P real-time PCR instrument employing the Brilliant II SYBR green QPCR Master Mix (Stratagene, La Jolla, CA) and the primers listed in Table 3. PCR reaction conditions included an initial 95°C step (10 min) followed by 40 cycles of 95°C (30 sec), 60°C (1 min) and 72°C (1 min).

### Statistical analysis

Statistical analysis of QPCR data was done using Student's *t* test after testing for differences of variances. *P*-values were considered significant, highly significant or extremely significant when <0.05, <0.01 or <0.001, respectively. Data represent means ± standard deviation (SD).
